# A novel gene signature predicts chemoradiotherapy efficacy and tumor immunity in high‐grade glioma

**DOI:** 10.1002/ctm2.170

**Published:** 2020-09-17

**Authors:** Shengchao Xu, Lu Tang, Gan Dai, Chengke Luo, Zhixiong Liu

**Affiliations:** ^1^ Department of Neurosurgery Xiangya Hospital of Central South University Changsha Hunan China; ^2^ Department of Thoracic Surgery Xiangya Hospital of Central South University Changsha Hunan China; ^3^ Department of Microbiology Xiangya School of Medicine, Central South University Changsha Hunan China

**Abbreviations**
AUC: area under curve
CGGA: Chinese Glioma Genome Atlas
CTLA‐4: cytotoxic T‐lymphocyte‐associated protein 4
ESTIMATE: Estimation of Stromal and Immune cells in Malignant Tumor tissues using Expression data
HGG: high‐grade glioma
LAG3: lymphocyte activation gene 3
LASSO: Least absolute shrinkage and selection operator
OS: overall survival
PD‐1: programmed death‐1
ROC: receiver operating characteristic
TIM‐3: T cell immunoglobulin and mucin domain 3
TIMER: Tumor Immune Estimation Resource
Vif: variance inflation factor

Dear Editor,

High‐grade glioma (HGG), defined as grade 3 and grade 4 glioma, is the most malignant brain cancer with high mortality.^1^ Although current management including maximal surgical resection, chemotherapy, and radiotherapy has achieved great advancement, the prognosis of patients with HGG remains unfavorable. The median overall survival (OS) of patients with grade 3 glioma is 3 years, whereas that of grade 4 glioma is 13 months.^2^ Besides, drug resistance impairs the prognosis of HGG patients and its mechanism remains unclear.^3^ Here we conducted this study to profile genes associated with sensitivity to chemoradiotherapy in patients with HGG. Moreover, a novel risk signature was constructed and its relationship with tumor microenvironment was identified. The primary objective was to screen predictive genes of chemoradiotherapy efficacy and tumor immunity in the treatment of HGG.

A total of 475 patients with HGG extracted from CGGA database were enrolled in our study. The RNA‐seq data and clinical characteristics of enrolled patients were obtained. Among these patients, there were 286 males and 189 females; 216 had grade 3 glioma and 259 had grade 4 glioma; the mean age was 45.6 ± 12.4 years; all patients received standard therapy including surgical resection, chemotherapy, and radiotherapy (Table S1). Patients were divided into high and low OS groups with the mean OS as the cut‐off value. Then we applied differential gene expression analysis to profile genes related to the efficacy of chemoradiotherapy. LASSO analysis was used to identified genes with predictive value. The selected genes were enrolled in multivariate Cox regression analysis to construct the risk model. Variance inflation factor (Vif) and hypothesis testing were used to filter out genes with high collinearity. After the construction of risk signature, we further explored the association between risk score and immune infiltration, which was calculated by ESTIMATE^4^ and TIMER algorithm.^5^

Totally, 128 differentially expressed genes with adjusted *P*‐value < .05 were identified including 74 upregulated and 54 downregulated genes (Figure 1A). The expression pattern was shown by heatmap (Figure 1B). LASSO analysis screened 17 genes and four genes (*MYT1L, LOC100133039, PPP1R1B, ABCC6*) were selected by multivariate Cox analysis for the construction of the risk model. The risk score ranged from 0.021 to 2.120 with the cut‐off value of 0.346; high risk and low risk group included 164 and 307 patients, respectively (Figure 1D). Survival analysis revealed that patients in high risk group enjoyed a poor prognosis (Figure 1E). Heatmap showed that the expression of four genes was higher in high risk group (Figure 1F). Notably, the four genes were down‐regulated in patients with higher OS, indicating that these genes were correlated with the sensitivity of chemoradiotherapy and prognosis in patients with HGG. Further we performed time‐dependent receiver operating characteristic (ROC) curve to evaluate the predictive value of risk score. Results showed that the area under curve (AUC) of 5‐year survival was 0.796, whereas 1‐year AUC was 0.659 and 3‐year AUC was 0.754, indicating that risk score had a promising predictive value for predicting long‐term survival in HGG (Figure 1G). These findings suggested that the gene signature was associated with clinical prognosis and had a good predictive value.

**FIGURE 1:**
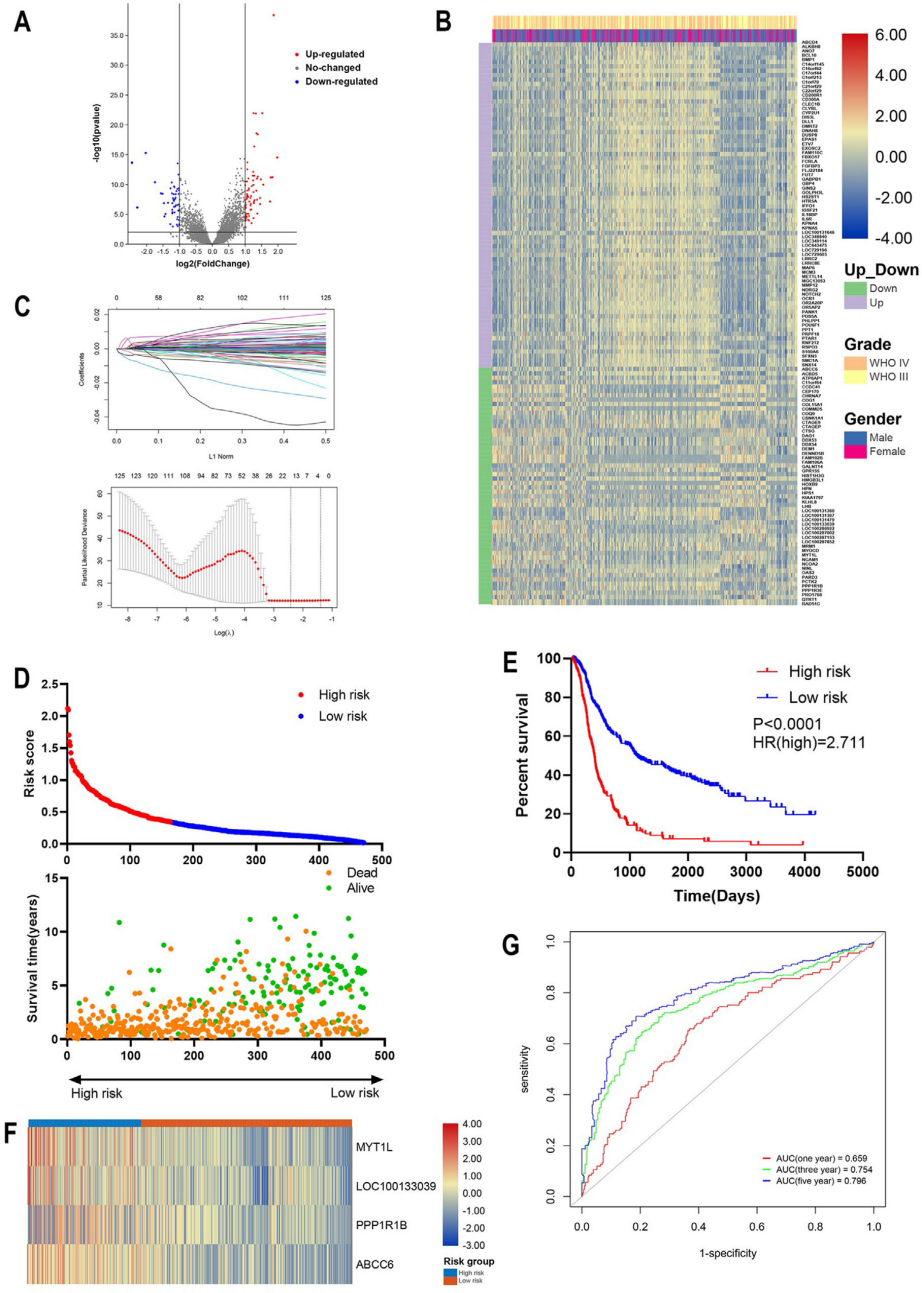
Identification of chemoradiotherapy associated genes. A, Up‐ and down‐regulated genes were identified in HGG patients received chemoradiotherapy. B, Heatmap showed the expression pattern of 128 differentially expressed genes in CGGA dataset. C, LASSO analysis selected predictive genes for the establishment of risk signature. D, Risk score distribution, survival status, and survival time of enrolled patients. E, Kaplan‐Meier analysis of high and low risk groups. F, Expression pattern of four selected genes in high and low risk groups. G, Time‐dependent ROC analysis of predictive value of risk score in HGG survival. Abbreviations: HGG, high‐grade glioma; LASSO, least absolute shrinkage and selection operator; ROC, receiver operating characteristic

Further, we employed ESTIMATE algorithm to evaluate the immune, stroma, and ESTIMATE score as well as tumor purity in enrolled samples. Results showed that immune, stroma, and ESTIMATE scores were significantly higher in high risk group whereas tumor purity was lower, indicating that risk score was associated with tumor microenvironment (Figure 2A). Moreover, risk score had a positive correlation with immune checkpoints including CTLA‐4, LAG3, TIM‐3, PD‐1, and CD80 (Figure 2B). LAG3 was proved to negatively regulate the activity of T cell and strongly promote the depletion of CD8^+^ T cell.^6^ The strong correlation between risk score and LAG3 might account for the bad prognosis of patients in high risk group (Figure S1A). Further, we applied TIMER algorithm to determine the infiltration of six immune cells including dendritic cell, B cell, neutrophil, CD4^+^ T cell, CD8^+^ T cell, and macrophage (Figure 2C). However, the value of the risk score had a poor correlation with the abundance of immune cells (Figure S1B). Besides, the content of B cell, dendritic cell, neutrophil, and CD4^+^ T cell was significantly higher in high risk group (Figure 2D). Multivariate Cox proportion hazard model revealed that risk score, B cell, and dendritic cell were independent risk factors whereas CD8^+^ T cell was an independent protective factor for patients with HGG (Figure S1C). CD8^+^ T cell was the conductor of immune surveillance and directly induced the elimination of tumor cells, which accounted for its protective role in HGG.^7^ The correlation between risk score with immune checkpoints and immune infiltration indicated that the risk signature was associated with tumor microenvironment in HGG. Therefore, risk signature might be used to predict the efficacy of immunotherapy in patients with HGG, which required further studies.

**FIGURE 2:**
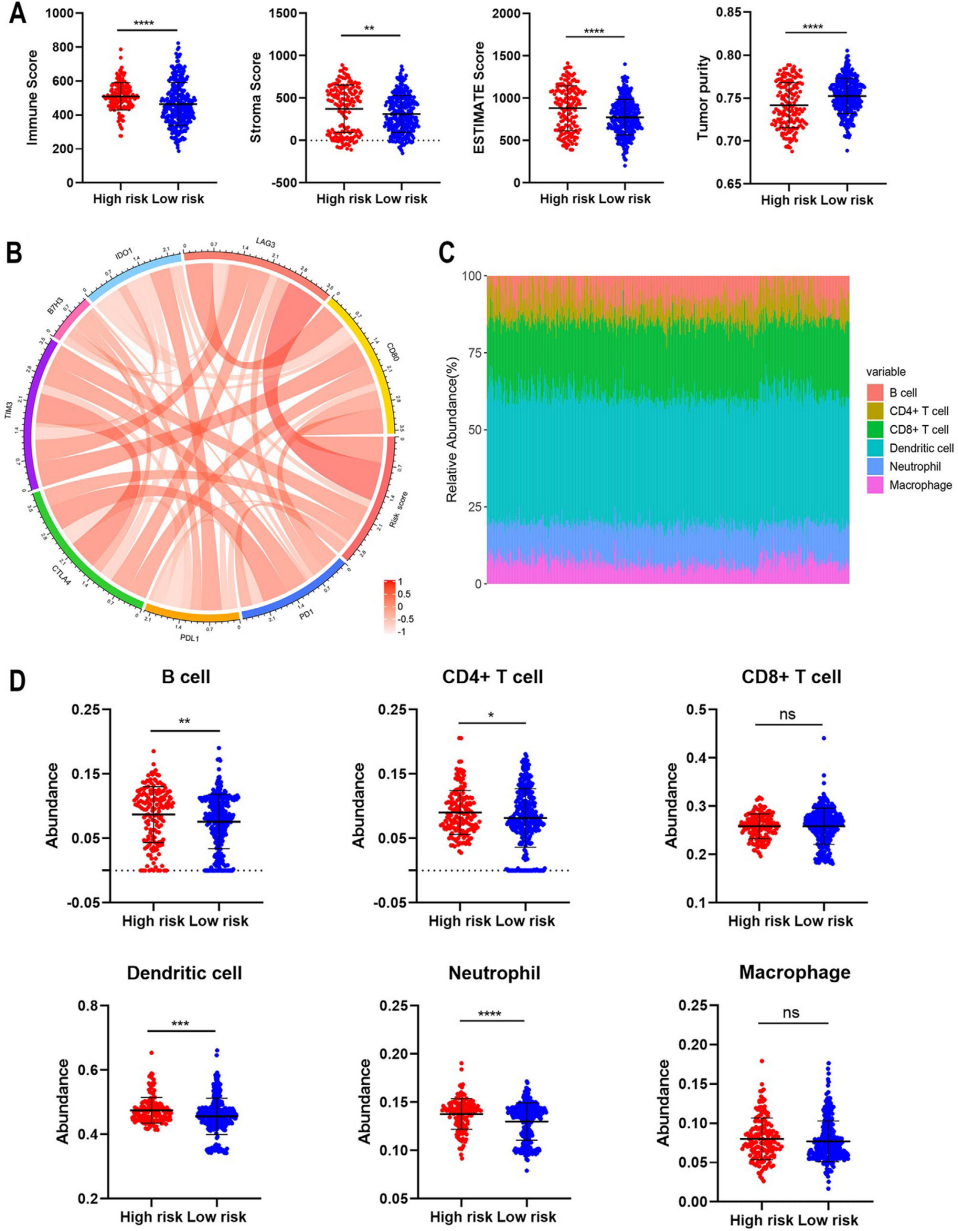
Association between risk signature with tumor microenvironment. A, The level of immune, stroma, and ESTIMATE score as well as tumor purity in high and low risk groups. B, Association between risk signature with immune checkpoints. C, Landscape of the abundance of six immune cells in microenvironment. D, The abundance of six immune cells in high and low risk groups. ^*^ *P* < .05; ^**^ *P* < .01; ^***^ *P* < .001; ^****^ *P* < .0001; ns, no significance. Abbreviation: ESTIMATE, Estimation of Stromal and Immune cells in Malignant Tumor tissues using Expression data

To sum up, we constructed a novel gene signature composed of four genes that were downregulated in patients with a better prognosis. The risk signature was markedly correlated with clinical prognosis, immune checkpoints, and immune infiltration. These findings indicated that the novel gene signature might be used to predict chemoradiotherapy efficacy and tumor immunity in HGG. Additional studies are required to determine the predictive value of risk signature in HGG immunotherapy.

## CONFLICT OF INTEREST

The authors declare no conflict of interest.

## Supporting information

Supporting

Supporting

## Data Availability

The data that support the findings of this study are available on reasonable request from the corresponding author.
