# Supplementary figures and images for: A novel gene signature predicts chemoradiotherapy efficacy and tumor immunity in high‐grade glioma
Source: Clin Transl Med. 2020 Sep 17;10(5):e170. doi: 10.1002/ctm2.170 (PMC7507345; doi:10.1002/ctm2.170)

A

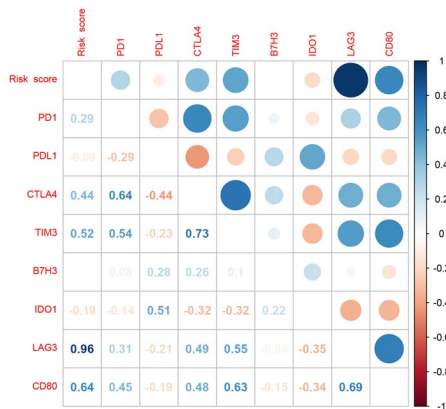

B

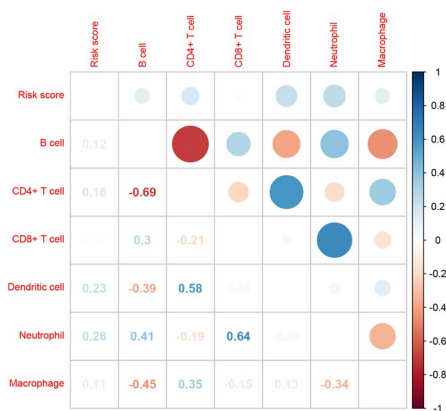

C

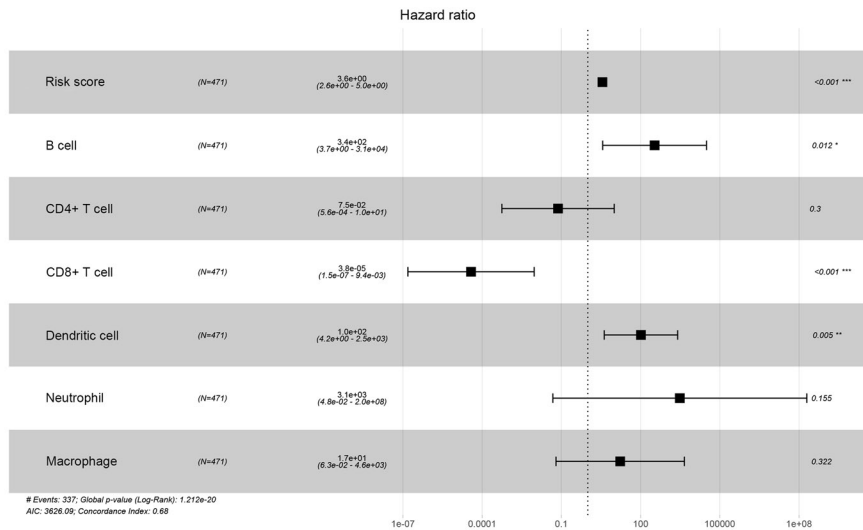

Supplement: Supplementary file 1 — Supporting [file CTM2-10-e170-s001.pdf]
